# Monoamine oxidases are mediators of oxidative stress in human varicose Veins: interactions with obesity, inflammation, and angiotensin II

**DOI:** 10.1007/s11010-025-05398-6

**Published:** 2025-09-29

**Authors:** Sonia Raţiu, Adrian Sturza, Paul S. Muntean, Claudia Borza, Tiberiu Bratu, Danina M. Muntean

**Affiliations:** 1 Doctoral School Medicine, “Victor Babeș” University of Medicine and Pharmacy of Timişoara, E. Murgu Square, Noo. 2, 300041 Timişoara, Romania; 2 Center for Translational Research and Systems Medicine, “Victor Babeș” University of Medicine and Pharmacy of Timişoara, E. Murgu Square, Noo. 2, 300041 Timişoara, Romania; 3 “Victor Babeș” University of Medicine and Pharmacy of Timişoara, E. Murgu Square, Noo. 2, 300041 Timişoara, Romania; 4 Department of Balneology, Medical Rehabilitation and Rheumatology – Clinic of Medical Rehabilitation, “Victor Babeș” University of Medicine and Pharmacy of Timişoara, E. Murgu Square, Noo. 2, 300041 Timişoara, Romania

**Keywords:** Chronic venous disease, Varicose veins, Monoamine oxidases A and B, Obesity, Oxidative stress, Inflammation, Angiotensin II

## Abstract

Chronic venous disease (CVD) and its clinical manifestation, the varicose veins (VVs), are characterized by progressive structural and functional alterations of the venous walls, with obesity/overweight being one of the most frequent comorbidities. Monoamine oxidases (MAO-A and MAO-B) are mitochondrial flavoenzymes responsible for the constant generation of hydrogen peroxide (H_2_O_2_) during the catabolism of biogenic monoamines and neurotransmitters that contribute, when upregulated, to the oxidative stress in most mammalian tissues. However, their role in the VV pathophysiology and its modulation by vasoactive stimuli such as angiotensin II (Ang II) remains unclear. This exploratory study was double-aimed i) to assess MAO expression in human VV samples in relation to obesity and systemic inflammation and ii) to determine the impact of pharmacological MAO inhibition on oxidative stress under basal and Ang II–stimulated conditions. To this aim, 20 patients with VV and an indication for cryostripping were randomized according to their body mass index (BMI) into obese (n = 10) and non-obese (*n* = 10) groups, and VV samples were harvested and used to assess the following: i) MAO-A and MAO-B gene expression by qRT-PCR, as well as expression and localization by immunofluorescence, and ii) H_2_O_2_ by means of the ferrous xylenol orange oxidation (FOX) assay. Furthermore, the effect of selective MAO inhibition (clorgyline 10 μM for MAO-A, selegiline 10 μM for MAO-B) was tested ex vivo both at baseline and following acute stimulation with Ang II (100 nM). We showed that both MAO-A and MAO-B are constitutively expressed in the human venous walls, with higher levels in the varicose veins than in the adjacent perforator veins. The obese patients with inflammatory status (elevated serum C-reactive protein) had significantly increased MAO-A (but not MAO-B) expression as compared to the non-obese controls (*p* < 0.01). Acute ex vivo incubation with Ang II further enhanced the expression of both isoforms and increased H_2_O_2_ generation. MAO inhibition significantly mitigated the oxidative stress in both non-stimulated and Ang II–stimulated samples, regardless of the presence or absence of obesity. In conclusion, MAO isoforms, in particular MAO-A, are upregulated in the human varicose veins and can be further induced by Ang II, especially in the setting of obesity associated with low-grade inflammation. MAO contributed to the local oxidative stress, which was significantly reduced by its pharmacological inhibition with MAO-A and B inhibitors, thus pointing to MAO as a potential therapeutic target in patients with CVD.

## Introduction

Chronic venous disease (CVD) is a highly prevalent pathology of the general population worldwide, being strongly associated with obesity/overweight, among other risk factors [1, 2]. The Clinical-Etiology-Anatomy-Pathophysiology (CEAP) classification, a standardized, internationally accepted classification system based on the clinical (C) manifestations and disease etiology (E), anatomy (A), and pathophysiology (P), is currently used for both clinical practice and scientific research reports on CVD [3]. Obesity, a chronic multisystem disease whose burden has surged at an accelerated rate in the past two decades [4], is characterized by central and ectopic fat distribution and several adverse health outcomes [5]. A seminal study reported a significant correlation between obesity/overweight and the CEAP C categories of CVD, which were independent of age and sex [6].

CVD class CEAP C2 corresponds to the presence of varicose veins (VVs), which are the earliest clinical manifestation (with or without symptoms) of chronic venous insufficiency (CVI) [7]. Primary VVs are dilated, tortuous superficial veins resulting from chronic increase in local venous pressure, dilation, reflux, and valve dysfunction, leading to structural and functional alterations of the venous walls [8]. The pathogenesis of VV formation is multifactorial and involves the dynamic interplay of hemodynamic/shear stress, chronic inflammation, oxidative stress, and extracellular matrix remodeling [9–11].

Oxidative stress, defined as the imbalance between increased reactive oxygen species (ROS) and decreased antioxidant defense, is a major contributor to vascular pathophysiology. Several vascular sources of ROS have been systematically investigated over time in numerous experimental and clinical settings, the most important being: NADPH oxidases (NOX), uncoupled and inducible endothelial nitric oxide synthases (eNOS, iNOS), xanthine oxidase (XO), and, more importantly, the dysfunctional mitochondria [12–15]. In recent years, monoamine oxidases (MAO), flavoenzymes anchored to the outer mitochondrial membrane, have been increasingly recognized as important contributors to the cardiovascular oxidative stress in various animal models of disease and clinical pathologies [16, 17], including those associated with obesity [18] and chronic inflammation [19, 20]. MAO exists in two isoforms, MAO-A and MAO-B, and its physiological role is to catalyze the inactivation of neurotransmitters and biogenic monoamines (e.g., norepinephrine, dopamine, serotonin/5-hydroxytryptamine, etc.) in most human organs/tissues [21]. The isoforms have different tissue distributions, substrate specificities, and inhibitor sensitivities. Accordingly, MAO-A is the major isoform found in cardiomyocytes, the cells of the arterial wall, adipocytes, and catecholaminergic neurones, whereas MAO-B is primarily located in platelets, glial cells, and serotonergic neurones. MAO isoforms exhibit substrate specificity as follows: MAO-A shows a high affinity for norepinephrine and 5-HT, MAO-B preferentially catabolizes phenylethylamine and benzylamine, while both isoforms oxidize dopamine and tyramine [22, 23]. The reaction of oxidative deamination constantly yields three reactive metabolites: hydrogen peroxide (H₂O₂), ammonia, and the corresponding aldehyde. Among these deleterious ancillary products, H₂O₂ has been mostly investigated as a contributor to the oxidative stress, including the vascular pathologies associated with increased MAO activity and/or expression. As such, the contribution of MAO to the vascular oxidative stress in various arterial beds, such as mammary arteries [24, 25], mesenteric artery branches [26, 27], and brachial artery collaterals [28] isolated from patients with cardio-metabolic and renal pathologies has been reported.

The overall role of oxidative stress in CVD has been recently reviewed [29, 30]. Excessive reactive oxygen species (ROS) generation in the venous walls contributes to the endothelial dysfunction, which, together with the changes in smooth muscle cell contractile phenotype, disturbances in collagen types secreted by fibroblasts, and local infiltration with inflammatory cells that release numerous proinflammatory cytokines, leads to the irreversible damage of the venous microcirculation [31]. Despite significant progress, the ROS sources responsible for the local oxidative stress in the venous wall are partially characterized. In particular, the role of MAO in the venous pathologies has been less explored. The vast majority of information regarding the roles of MAO in the catabolism of biogenic amines in the animal venous system comes from the pioneering work carried out in the 80 s by the group of W. Osswald. In the early 90 s, these authors assessed the activities of MAO-A and B in saphenous vein segments harvested from patients undergoing coronary bypass surgery and reported the presence of both isoforms in the healthy human veins and their role in the deamination of exogenous tritiated noradrenaline [32, 33]. To the best of our knowledge, MAO expression in the human varicose veins has not been assessed so far.

Shifting gears, it has been reported that angiotensin II (Ang II) elicited the activation of MAO-A in HL-1 cardiomyocytes [34] and mitral valve explants harvested from patients with severe mitral regurgitation and indication for surgical repair [35], an effect mediated in both cases via the AT1 receptors. Ang II has a crucial role in promoting vascular inflammation, oxidative stress, and remodeling that has been systematically demonstrated in the arteries [36, 37], while being less explored in the veins. Of note, there is one study in the literature that reported an early reduction of Ang II apparent affinity in VV harvested from patients with CEAP C2 CVD and decreased contractility to Ang II in the more advanced stages of the disease [38].

The present exploratory study was double-aimed i) to assess MAO expression in human VV in relation to obesity and systemic inflammation and ii) to determine the impact of ex vivo MAO inhibition on venous oxidative stress under basal and Ang II-stimulated conditions.

## Material and methods

### Study groups

A total of 20 patients hospitalized at the First University Clinic of Surgery from”Pius Brînzeu’’ Emergency County Hospital of Timișoara, Romania, undergoing surgical treatment for primary varicose veins (CEAP C2s), were enrolled and randomized into two groups according to the body mass index (BMI): 10 obese patients (BMI ≥ 30 kg/m^2^, OB group, 1 M, 9 F) and 10 non-obese patients (BMI < 30 kg/m^2^, NON-OB/CTRL group, 2 M, 8 F). The study protocol was reviewed and approved by the Ethics Committee for Scientific Research of the “Victor Babeș” University of Medicine and Pharmacy, Timișoara, Romania (approval no. 62/17.12.2020). All participants provided written informed consent prior to inclusion according to the principles of the Declaration of Helsinki and relevant institutional guidelines (including the agreement for the use of the results for scientific purposes).

### Tissue collection and preparation

All patients were diagnosed with CVD CEAP C2 or C3 and underwent a standard surgical treatment for varicose veins, i.e., the cryostripping technique. During the intervention, segments of varicose veins (great saphenous vein) and adjacent perforator veins were excised, placed in Hanks’ solution on ice, and transported to the Center for Translational Research and Systems Medicine where they were prepared for further analysis (either frozen or immediately used). In some experiments, the samples were incubated at 37℃ in endothelial cell growth basal medium (EBM) containing 0.1% bovine serum albumin (BSA), in the presence or absence of angiotensin II (Ang II, 100 nM) and MAO inhibitors (clorgyline 10 μM and selegiline 10 μM) for a 12 h duration. These concentrations were selected based on our previous studies performed in arterial samples where an increased MAO gene and protein expression was reported after acute ex vivo incubation.

### Assessment of MAO gene expression by quantitative real-time PCR

Gene expression of both MAO isoforms in the VV samples was assessed as previously described [39, 40]. In brief, after tissue homogenization with the Tissue Lyser (Qiagen), total RNA was extracted utilizing the Aurum Total RNA Mini Kit (Bio-Rad), and concentration was assessed by means of a spectrophotometer (Nanodrop 2000, Thermo Scientific). Reverse transcription was performed with the iScript Advanced cDNA Synthesis Kit (Bio-Rad). Quantitative real-time PCR (qRT-PCR) was carried out with the CFX Connect Real-Time equipment (Bio-Rad) using the primers designed from NCBI reference sequences for human MAO-A (fw: CTG ATC GAC TTG CTA AGC TAC; rev: ATG CAC TGG ATG TAA AGC TTC) and MAO-B. The housekeeping gene EEF2 (fw: GAC ATC ACC AAG GGT GTG CAG; rev: GCG GTC AGC ACA CTG GCA TA) was used as the internal reference. Relative gene expression was calculated using the 2^–ΔΔCt method.

### Assessment of MAO expression and localization by immunofluorescence

MAO-A and MAO-B proteins were evaluated using indirect immunofluorescence on frozen venous tissue Sects. (5 µm), as previously described [28]. Samples were incubated overnight at 4℃ with the primary antibodies against MAO-A (Abcam, ab126751) and MAO-B (Abcam, ab175136), respectively. Alexa Fluor-labeled secondary goat anti-rabbit antibody (Invitrogen, A32731) was applied, and nuclei were counterstained with DAPI (Santa Cruz, SC3598). Slides were examined using a confocal fluorescence microscope (Olympus Fluoview FV1000), and a semi-quantitative analysis of fluorescence intensity was performed using the ImageJ software.

### Measurement of reactive oxygen species (ROS) production

Venous tissue homogenates were prepared in phosphate-buffered saline (PBS), and ROS generation was assessed using the ferrous iron oxidation (FOX) assay according to an initially established protocol [41], which quantifies the tissue level of hydroperoxides. The principle of the assay is that peroxides oxidize ferrous to ferric iron, which forms a colored compound with xylenol orange, whose absorbance was measured at 560 nm using a spectrophotometer (Jenway 6100). H_2_O_2_ generation was calculated using a standard curve; the results were expressed as nmol H₂O₂/h/mg of tissue.

### Statistics

Statistical analyses were conducted using GraphPad Prism, version 9.3.1 (GraphPad, USA). Data are expressed as mean ± SEM and were compared using one-way ANOVA. A p-value < 0.05 was considered statistically significant.

## Results

Baseline demographic characteristics and the main clinical and biochemical parameters are summarized in Table 1.

**Table 1:** Characteristics of the study groups

Parameter	NON-OB (CTL) (n = 10; 2♂, 8♀)	OB (n = 10; 1♂, 9♀)	p
BMI (kg/m^2^)	**24.66 ± 0.8**	**34.62 ± 1.75**	*
Age (y)	51.2 ± 3.76	53.2 ± 3.7	ns
ESR (mm/h)	**9 ± 1.08**	**15.5 ± 2.12**	*
C-reactive protein (mg/L)	**1.24 ± 0.26**	**6.41 ± 1.4**	*
Fasting blood glucose (mg/dL)	94 ± 3.21	110.9 ± 8.48	ns
Urea (mg/dL)	31.6 ± 2.529	37.7 ± 3.9	ns
Creatinine (mg/dL)	0.74 ± 0.04	0.81 ± 0.05	ns
Uric acid (mg/dL)	4.01 ± 0.34	4.91 ± 0.44	ns
Total cholesterol (mg/dL)	**209 ± 9.84**	**180 ± 12.27**	*
HDLc (mg/dL)	64.41 ± 3.12	57.8 ± 8.67	ns
LDLc (mg/dL)	**121.6 ± 9.16**	**103.4 ± 11.46**	*
Triglycerides (mg/dL)	**65.6 ± 9.65**	**102.5 ± 17.40**	*
ALAT (U/L)	**24.33 ± 3.17**	**37.4 ± 7.64**	*
ASAT(U/L)	**20.2 ± 2.3**	** ± 3.1**	ns

^*^ < 0.05

### Patients in the obese group had an inflammatory status and metabolic impairment

Twenty patients undergoing surgery for primary VV were randomized into two groups (*n* = 10/group) by BMI: non-obese (< 30 kg/m^2^) and obese (≥ 30 kg/m^2^). While age and gender distribution were comparable, the obese group had significantly higher ESR and CRP levels, as hallmarks of the inflammatory status. Lipid profile showed lower total and LDL cholesterol (due to the statin therapy), but significantly higher triglycerides in obese patients (reflecting metabolic impairment and/or unhealthy dietary habits). While kidney function parameters (creatinine, urea, uric acid) did not differ significantly, fasting blood glucose was higher in the obese group, suggesting a trend toward impaired glucose regulation. From the liver enzymes, particularly ALAT was elevated, indicating possible subclinical hepatic steatosis. Taken together, these results demonstrate the presence of systemic inflammation, impaired metabolism, and mild liver dysfunction in CVD patients from the OB *vs* NON-OB (CTL) group.

### MAO-A and MAO-B isoforms are constitutively expressed in the diseased human veins

**Fig. 1:**
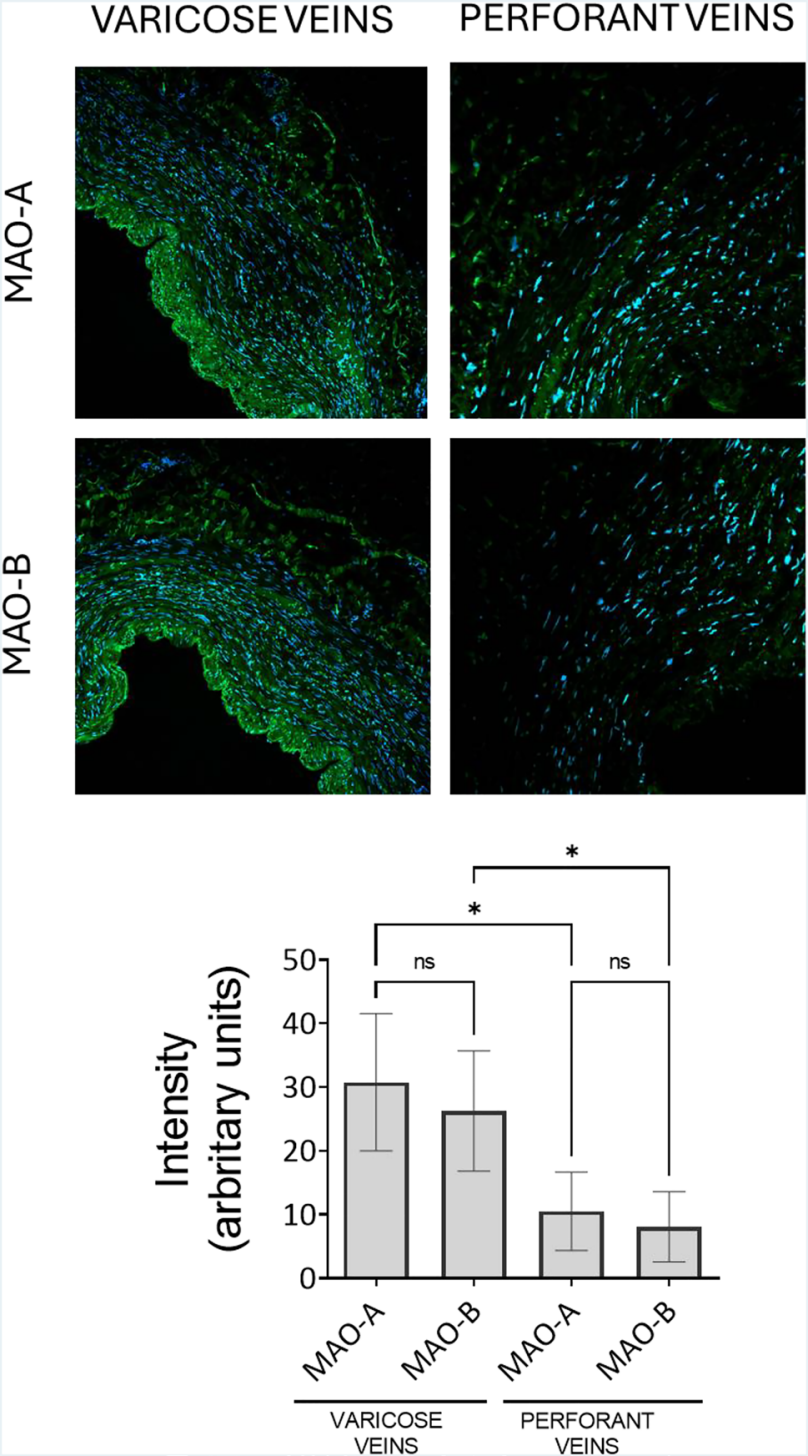
Expression of MAO-A and MAO-B (green) in the varicose veins and perforant veins. DAPI (blue). *n* = 10, *p < 0.05

Immunofluorescence experiments showed the constitutive expression of both MAO-A and MAO-B in the walls of varicose veins of both groups of CVD patients (Fig. 1). MAO immunoreactivity was localized to the endothelium, tunica media, and adventitia. Moreover, as shown in Fig. 1, MAO expression was significantly increased in the varicose veins as compared with the adjacent perforator veins from the same patient, pointing to a role of impaired hemodynamics in the localized MAO upregulation that will contribute to oxidative stress and possibly to the structural changes.

### MAO expression is significantly elevated in obese patients with systemic inflammation

**Fig. 2:**
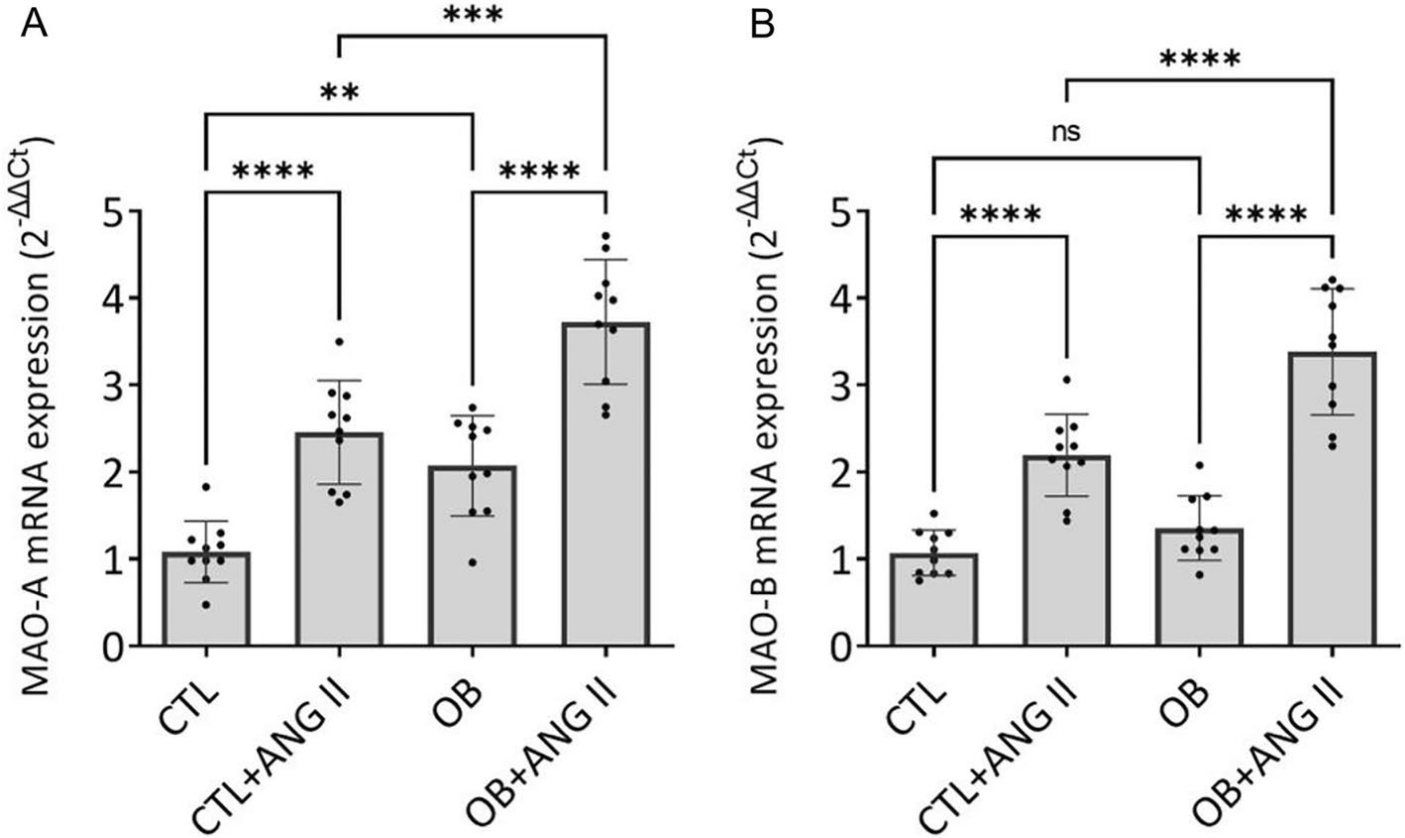
The effect of Ang II on the mRNA expression of MAO isoforms in varicose vein samples from obese (OB, n = 10) *vs*. non-obese (CTL, n = 10) patients. **p < 0.01, ***p < 0.001, ****p < 0.0001

The qRT-PCR results confirmed the presence of gene expression in the walls of the VV for both MAO-A (Fig. 2A) and MAO-B (Fig. 2B), suggesting a shared role in local monoaminergic metabolism and redox regulation.

Patients from the CTL group (BMI < 30 kg/m^2^) had comparable baseline levels for both isoenzymes. Patients with obesity (BMI > 30 kg/m^2^) and systemic inflammation (CRP > 5 mg/L) displayed significantly higher levels of MAO-A (p < 0.01) in their VV as compared to the non-obese controls (Fig. 2A). At variance, the baseline expression for MAO-B was similar in both groups (Fig. 2B).

Acute stimulation of the VV samples with Ang II (100 nM) led to a marked (*p* < 0.0001) comparable increase in the expression of both MAO-A (Fig. 2A) and MAO-B (Fig. 2B). This result indicates that constitutive MAO expression is responsive to vasoactive stimuli, pointing to a mechanistic link between local neurohumoral activation and oxidative stress within the venous wall. Taken together, these findings implicate MAO upregulation, particularly of the MAO-A isoform, as a potential contributor to venous oxidative stress and disease progression.

### Pharmacological inhibition of MAO attenuated oxidative stress in human varicose veins

**Fig. 3:**
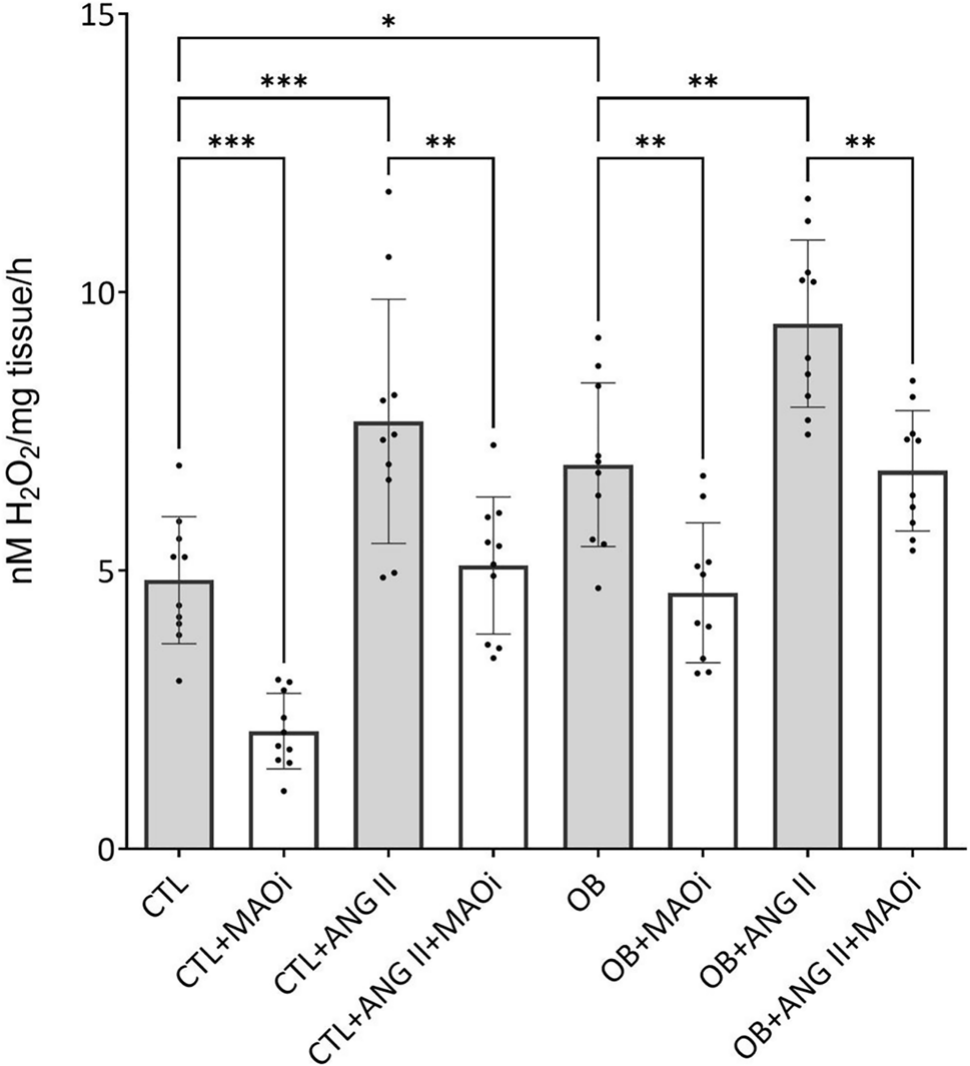
Evaluation of the venous H_2_O_2_ generation in obese (OB) and non-obese (CTL) patients after incubation (12 h) of the VV with Ang II (100 nM) and MAO inhibitors (clorgyline, 10 μM) and selegiline (10 μM), *p < 0.05, **p < 0.01, ***p < 0.001

Acute ex vivo incubation (12 h, 37℃) of the VV samples with selective MAO inhibitors— clorgyline (MAO-A selective, 10 μM) and selegiline (MAO-B selective, 10 μM) significantly reduced oxidative stress assessed by the H₂O₂ levels (FOX assay) (Fig. 3). Baseline H₂O₂ levels were significantly elevated (*p* < 0.05) in the obese VV samples compared to controls, reflecting an already increased oxidative stress. Ang II stimulation further increased H₂O₂ production in both CTL and OB groups; the levels were higher in obese patients, albeit not reaching statistical significance. Importantly, treatment with MAO inhibitors significantly attenuated (*p* < 0.01) oxidative stress under all samples, both non-stimulated and stimulated with Ang II (Fig. 3). These results demonstrate that MAO-A and B isoforms contribute to ROS generation in the varicose veins, especially in the setting of obesity when induced by pro-oxidant stimuli such as Ang II. Pharmacological inhibition of MAO effectively reduces H₂O₂ production, highlighting MAO as a potential therapeutic target to mitigate oxidative damage in patients with CVD, regardless of the presence or absence of obesity.

## Discussion

The main finding of this exploratory study is that monoamine oxidases, traditionally studied in the pathogenesis of neurodegenerative/psychiatric disorders and more recently in cardiovascular pathologies, are significant contributors to oxidative stress in human varicose veins harvested from patients with CVD. The greater abundance of both isoforms in the varicose veins compared to adjacent perforator veins suggests that their upregulation is a localized phenomenon rather than a diffuse alteration. Such a pattern is consistent with the concept that changes in shear stress, together with the increased venous pressure, reflux, and stasis, induce chronic inflammation, oxidative stress, and ultimately, remodeling of the venous walls and valves [42]. We found that both MAO-A and MAO-B were detected in the endothelial, medial, and adventitial layers of the VV. At variance, a seminal paper published more than two decades ago reported that both human arteries and veins presented MAO-A and MAO-B immunoreactivity in the muscular layers and fibroblasts (but not in endothelial cells) [43]. We also showed that the MAO-A isoform displayed an increased basal expression in obese *vs* non-obese patients with CVD. This observation is in line with our findings in the arterial bed. As such, we reported that MAO-A was upregulated in the mesenteric artery branches isolated from obese patients subjected to elective abdominal surgery [27] and mammary arteries harvested from overweight patients subjected to coronary revascularization [25]. The presence and role of MAO in the venous circulation were scarcely addressed in the literature. Pioneering studies by Caramona in the early 80 s accurately described the localization (intra- and extraneuronal) of MAO-A and B in the dog saphenous vein [44, 45] and elegantly demonstrated the role of the MAO-A (but not MAO-B) isoform in the inactivation of noradrenaline in the canine venous system [46]. This group also firstly quantified the activities (but not the location) of MAO-A and B in intact human saphenous vein samples used for coronary bypass surgery and showed that exogenous noradrenaline is deaminated by both MAO-A and MAO-B [32]. Further studies are required to assess the intra- and extraneuronal location of the MAO isoforms in human healthy and diseased veins.

We have assessed herein that H_2_O_2_ production in the varicose veins and showed that it can be mitigated by MAO inhibition in both CTL and obese patients. Nowadays, the physiological roles of H_2_O_2_ as a second messenger that regulates several processes relevant to both CVD and obesity, such as cellular proliferation, wound healing, insulin signaling, etc., are widely acknowledged [47]. While there is an ongoing interest in the study of oxidative stress in cardiovascular pathologies [48], there is an unmet need for identifying the right moment for treating diseases associated with oxidative stress while preserving physiological ROS formation [49, 50]. Since most of the clinical trials with antioxidant therapies were disappointing, there is an unmet need for pharmacologically targeting upregulated/dysfunctional enzymatic sources of ROS, including MAO [51].

We also showed herein that basal oxidative stress was higher in the VV from the obese group with systemic inflammation, as shown by the elevated plasma level of C-reactive protein. This observation is consistent with the role of inflammatory cytokines in driving mitochondrial oxidative enzyme upregulation and subsequent oxidative stress. In line, we have reported that acute ex vivo incubation of mesenteric artery branches with IL-6 significantly increased MAO-A gene expression [20], supporting the hypothesis of oxidative stress driven by inflammation. Similarly, we have recently reported a significantly increased expression of iNOS (*p* < 0.001) and the subsequent increased ROS level (p < 0.05) in VV from the obese patients with inflammatory status [40].

In concert with the reports in the arterial system, it is tempting to speculate that inflammation may act as a direct activator of mitochondrial oxidative pathways already in the early stages (CEAP C2) of obese/overweight CVD patients. The “oxidative response to inflammation” hypothesis in the pathogenesis of ATS has been postulated two decades ago by Stockey and Keaney, who speculated at that time on the failure of the antioxidant strategies, arguing that oxidative stress is rather the consequence, not the cause, of ATS [52]. Accordingly, the “normal-to-injury” phenotype switch of the vascular endothelium is the trigger event for the recruitment of inflammatory cells and margination to the arterial wall, resulting in vascular inflammation. The inflammatory process is pivotal in the development of the vascular lesions and triggers ROS (also, reactive nitrogen species) generation, leading to oxidative (and nitrosative) stress, which modulates the characteristics of ATS plaques, although not directly causing ATS. However, it has to be mentioned that the process of chronic vascular inflammation, which has been extensively studied in relation to ATS, has also been linked to the hypoxia-driven signaling cascades rather than the oxidative stress [53, 54]; this might be relevant for the diseased veins, since hypoxia also occurs mainly in the advanced CVD. An elegant study has demonstrated that endothelial cells are able to autonomously synthesize catecholamines in response to hypoxia by upregulating the genes encoding the enzymes involved in their synthesis and activating the corresponding signal transduction pathways [55]; whether providing the MAO substrates in the venous endothelial cells will activate the enzyme remains to be investigated. Importantly, it has been reported that chronic intermittent hypoxia significantly increased MAO-A expression in the rat hippocampus with subsequent oxidative stress and NF-κB-mediated inflammation [56], pointing to the occurrence of the “inflammation response to the oxidative” environment, as proof of the bidirectional crosstalk between these two major pathomechanisms. It is tempting to further speculate that the venous ROS production may contribute to a “ROS-induced ROS release” (RIRR), a phenomenon described in the heart [57] and arteries [58], whereby an initial oxidative burst triggers feed-forward mitochondrial ROS generation, creating a cascade of redox signaling and a self-perpetuating cycle of oxidative injury. Such feedback loops that perpetuate endothelial dysfunction and chronic inflammation may similarly underlie the progressive remodeling of venous walls observed in advanced chronic venous disease. The most studied signaling loop of RIRR involves the NADPH oxidases (Nox) family and mitochondria. As such, in HUVECs, Nox4-derived H_2_O_2_ can activate Nox2 to increase mitochondrial ROS generation (via p66Shc phosphorylation), which in turn will enhance VEGF signaling and angiogenesis [59]. The contribution of RIRR to the redox signaling in the venous walls requires further investigation.

The same holds true for the so-called process of “mito-inflammation,” which has been described in most non-communicable diseases [60]; whether this may also operate in CVD remains an open question.

Another key finding of the present study is that acute stimulation with Ang II significantly increased the venous expression of both MAO isoforms, further amplifying H_2_O_2_ generation in VV from obese and non-obese patients. The responsiveness of MAO to Ang II links neurohumoral activation to venous oxidative stress, suggesting that MAO may serve as a convergence point for metabolic, inflammatory, and vascular injury signals in CVD. This connection is particularly relevant in obese patients, who often have heightened renin–angiotensin system activity and low-grade systemic inflammation that both potentiate mitochondrial oxidative stress [61, 62]. We have to mention as a limitation of the study that we did not address herein the signal transduction of Ang II-mediated MAO induction. However, we have firstly demonstrated that acute incubation of isolated rat aortic rings with Ang II elicited an NF-κB-mediated MAO overexpression and that AT1 receptors inhibition blocked the Ang II-stimulated MAO-A and B induction [63]. The role of Ang II-mediated signal transduction has been extensively studied in relation to cardiac hypertrophy and the involvement of oxidative stress, in particular the activation of the Nox family [64]. Also, acute exposure of human coronary artery endothelial cells to moderate concentration Ang II (10 nM/L, 12 h) upregulated several genes implicated in blood vessel development and this pro-angiogenic effect involved the miR-21 dependent activation of the STAT3 signaling cascade [65]. By far less information is available regarding its role in signal transduction in the venous system. The group of Agrawal unequivocally demonstrated in an elegant study performed in human saphenous vein bypass conduits the role of Ang II in proliferation and migration of vascular smooth muscle cells. These authors showed that Ang II dose-and time-dependently increased the connexin 43 expression and subsequently the intercellular communication at the gap junctions via a signaling pathway that involved the AT1 receptor and the activation of mitogen-activated protein kinases [66]. Further research is required to elucidate the Ang II-mediated signal transduction in relation to MAOs in the setting of CVD.

Last but not least, from a therapeutic perspective, our findings that selective inhibition of MAO-A and MAO-B significantly reduced hydrogen peroxide production, both at baseline and under Ang II stimulation, are clinically meaningful. They indicate that MAO inhibition can blunt oxidative stress even in the presence of potent pro-oxidant triggers, interrupting not only the primary ROS generation but potentially also the secondary amplification mediated by the RIRR. Currently, MAO inhibitors are traditionally used for Parkinson’s disease and some forms of depression but are being explored for repurposing in other pathologies, in particular in various types of cancer [67, 68]. Given that novel, selective, and reversible MAO inhibitors are already in clinical use for neurological conditions, repurposing these agents for CVD could provide a novel targeted approach to mitigating venous oxidative injury.

Despite the insightful information this exploratory study offered, a number of limitations must be noted. These include the reduced sample size, the lack of addressing the signal transduction of MAO-related oxidative stress and of Ang II induction, as well as the pharmacological testing of only the irreversible MAO inhibitors. Another limitation of our study is the lack of assessment of insulin resistance using the homeostatic model assessment (HOMA-IR) index. Given that obesity is frequently associated with impaired glucose metabolism and insulin resistance, evaluating this parameter could have provided additional insights into the interplay between metabolic dysfunction, inflammation, and MAO-related oxidative stress in varicose veins. Nonetheless, the demonstration of MAO’s presence in the varicose veins, its association with obesity and inflammation together with its induction by Ang II, adds a novel dimension to the understanding of oxidative mechanisms in varicose veins and highlights MAO as a target for both mechanistic research and therapeutic innovation in the setting of chronic venous disease.

## Conclusions

Both MAO-A and MAO-B are constitutively expressed in the diseased veins of patients with CVD, with higher levels in the varicose segments as compared to adjacent perforator veins. Obesity and systemic inflammation are associated with a significant upregulation of MAO-A expression in varicose veins. Angiotensin II further enhanced the expression of both MAO-A and MAO-B and amplified hydrogen peroxide production, thereby linking local neurohumoral activation to the venous oxidative stress. Selective inhibition of MAO significantly reduced the tissue level of ROS both at baseline and during angiotensin II stimulation, highlighting the role of MAO as a promising therapeutic target in chronic venous disease.

## Data Availability

The authors confirm that data supporting the findings of this study are included within the article.
